# Acute Modulation of Circulating Exerkines Responses to a Circuit and Traditional Resistance Training in Young Adults: A Pilot Study

**DOI:** 10.3390/biom16060827

**Published:** 2026-06-02

**Authors:** Pragya Sharma Ghimire, Adam Eckart, Madhumitha Sadhasivan Gayathri, Michelle Manochio

**Affiliations:** College of Health Professions and Human Services, Kean University, 1000 Morris Ave, Union, NJ 07083, USA; eckarta@kean.edu (A.E.); sadhasim@kean.edu (M.S.G.); michelle.manochio@kean.edu (M.M.)

**Keywords:** exerkines, training protocol, musculoskeletal health, sex differences

## Abstract

Evidence suggests that physical activity promotes bone health through mechanical loading and biochemical signaling between bone and muscle tissues. A class of signaling molecules known as exerkines is a key mediator of bone–muscle crosstalk. Although exercise regulates osteokines, the acute exerkine responses across different exercise modalities remain unclear. This randomized repeated-measures crossover study compared acute changes in serum sclerostin (SCL), dickkopf-1 (DKK-1), receptor activator of nuclear factor kappa-B ligand (RANKL), osteopontin (OPN), brain-derived neurotrophic factor (BDNF), irisin, and interleukin 6 (IL-6) following circuit training (CT) (cycle ergometer, push-up, step-ups, medicine ball twist, and front squats with kettlebell for three rounds) and traditional resistance (TR) exercise (3 sets 10 repetitions 50–60% 1 RM for leg press, seated cable row, barbell bench press, dumbbell deadlifts, and dumbbell seated shoulder press) in healthy young adults (n = 12). Participants performed two protocols separated by 2-week wash-out periods. Blood samples were analyzed before exercise training (pre), immediately post-exercise (IP), and 30 min post-exercise (30P) for all exerkines using ELISA. There was a significant interaction between protocol, timepoint, and sex (*p* = 0.038) for SCL levels. In males, SCL levels increased from Pre to IP under both training protocols (CT: 0.10 ± 0.02 ng/mL to 0.14 ± 0.02 ng/mL; TR: 0.20 ± 0.02 ng/mL to 0.21 ± 0.02 ng/mL). In both protocols, SCL levels decreased from IP to 30 P (CT: 0.14 ± 0.02 to 0.10 ± 0.01 ng/mL; TR: 0.22 ± 0.02 to 0.17 ± 0.02 ng/mL). In females, SCL levels increased from Pre to IP under both training protocols (CT: 0.03 ± 0.02 ng/mL to 0.06 ± 0.02 ng/mL; TR: 0.07 ± 0.02 ng/mL to 0.13 ± 0.02 ng/mL). There was a significant time effect for OPN and RANKL concentrations. Marginal means for the time point showed that OPN was significantly higher at the Pre time point. Post hoc analyses showed that OPN levels significantly decreased from 30P to Pre (18.84 ± 0.92 to 15.69 ± 1.32 pg/mL) (*p* = 0.01). RANKL showed a significant increase from Pre (0.38 ± 0.04 pg/mL) to 30P (0.57 ± 0.06 pg/mL) (*p* = 0.02); otherwise, there were no significant differences between protocols or sexes. Irisin significantly decreased from Pre (28,761.73 ± 238.52 pg/mL) to IP (2364.85 ± 243.79 pg/mL) in both protocols (*p* = 0.01). DKK-1, BDNF, and IL-6 levels were only different between protocols (*p* < 0.01). SCL and BDNF levels were expressed higher in the TR protocol, whereas DKK-1, IL-6, and Irisin levels were expressed higher in the CT protocol. Overall, the findings suggest that SCL, RANKL, OPN, and irisin responded to the exercise bout, while the other exerkines did not show meaningful changes over time.

## 1. Introduction

Overwhelming evidence suggests that exercise plays a pivotal role in improving health outcomes, reducing the risk of chronic disease, and enhancing quality of life. In scientific disclosure, the terms “exercise” and “physical activity” are commonly used interchangeably, even though they represent conceptually distinct phenomena [1,2]. Exercise typically comprises structured aerobic, resistance, and high-intensity activities, whereas physical activity involves routine exercise, recreational, occupational, and household activities [2]. Although many scientific attempts have shown that regular exercise is beneficial, the underlying molecular mechanisms in providing benefits are not fully understood. Exercise is considered a non-pharmacological intervention, and recent research has shown that exercise-induced molecules, named “exerkines”, are a critical mediator associated with exercise and musculoskeletal health [3].

Exerkines are bioactive molecules released in response to exercise and collectively form an intricate signaling network that regulates exercise-induced adaptations across the metabolic, musculoskeletal, cardiovascular, and neural systems [4]. Biochemically, exerkines are peptides, nucleic acids, and microRNAs that act through autocrine, paracrine, and endocrine pathways to affect various organs and tissues. It is well established that mechanical loading is considered the primary driver of musculoskeletal adaptation [3]. The emerging evidence demonstrates that exercise-induced biochemical signaling, mediated by cytokines, myokines, and osteokines, further governs bone-muscle crosstalk. At the molecular level, muscle and bone share conserved mechanotransductive and signaling pathways, including Wnt/β-catenin, MAPK cascades, and PI3K/AKT/mTOR pathways. Major discoveries on exerkines such as sclerostin (SCL), dickkopf-1 (DKK-1), receptor activator of nuclear factor kappa-B ligand (RANKL), osteopontin (OPN), brain-derived neurotrophic factor (BDNF), interleukin 6 (IL-6), Tumor Necrosis Alpha (TNF-α), CX3CL-1, and irisin have highlighted their diverse functions in human health [3,5,6]. The wide variety of exerkines highlights how exercise affects many physiological processes and highlights their potential for therapeutic interventions in metabolic diseases and other health conditions [7,8]. However, the question remains: how do molecular mechanisms differ across exercise prescriptions, including acute and chronic bouts of exercise, and how are exerkines secreted?

Emerging evidence suggests that an acute bout of exercise triggers signaling cascades, whereas regular exercise acts as a remodeling regulator, recalibrating baseline endocrine function. Evidence shows that exerkine-mediated benefits can be driven by a single bout rather than chronic exercise training protocols [9]. Notably, exercise intensity and duration appear to be critical determinants of exerkine release. A meta-analysis has shown that acute endurance exercise significantly increases circulating myokines, and that the responses depend on the exercise dose–response relationship [10]. However, these findings predominantly reflect protocols that combine aerobic and resistance training modalities. Reciprocally, acute exercise has also shown a transient increase in exerkines of bone metabolism, reflecting a short-term shift toward an anabolic bone environment. These effects are regulated via bone–muscle crosstalk, whereby cytokines secreted by contracting skeletal muscle contribute to the regulation of bone homeostasis [11].

During an acute bout of exercise, it is important to recognize that these short-lived signaling events can serve as a foundation for adaptation and depend on the participant’s fitness level [9]. Among exercise types, circuit training has been shown to stimulate systems that promote physiological health. Circuit training (CT) is considered a versatile training modality because it requires less time than other modalities, concurrently enhancing cardiovascular and neuromuscular function. It is a structured alternation between aerobic and resistance-based movements within a single session. Additionally, circuit-based training modalities are associated with a relatively low risk of musculoskeletal injury, high exercise adherence, and minimal equipment requirements [12]. Although CT uses lower loads to stimulate bone mass adaptation than traditional resistance (TR) exercise, there is evidence that it is effective in addressing osteopenia and osteoporosis in older adults [13]. It should be noted that high-intensity training and traditional strength training programs ranked among the leading global fitness trends in 2025 by the American College of Sports Medicine, highlighting their relevance and value in the aging population [14].

Although the mechanisms of acute exercise across various physiological systems have been investigated, a notable gap remains in understanding the differences in the acute effects of CT and TR exercise protocols on exerkine responses. In this context, the study compared the acute effects of CT and TR training protocols, two in situ exercise models, on circulating exerkines. We hypothesized that an acute bout of exercise would elicit differential circulating exerkine responses between the CT and TR protocols due to their distinct mechanical and metabolic demands.

## 2. Materials and Methods

### 2.1. Participants

This study used a randomized, repeated-measures crossover design in which all participants completed two exercise protocols, CT and TR, in random order. G*Power version 3.1.9.7 analysis was used to estimate the sample sizes needed for 80% power based on α = 0.05 and effect sizes (Cohen’s d) for the sex differences in SCL and IL-6 [15]. The effect size (d) difference between the two protocols was determined to be 2.0, a large effect size requiring a sample size of 5 per group; however, due to the possibility of dropouts and outliers, a sample size of 6 per group was used for this study. A total of 18 participants aged 18–30 years were recruited for this study; 6 participants were lost during follow-up and were excluded; therefore, 12 healthy adults: men (n = 6) and women (n = 6) of diverse ethnicities (Caucasian, African American, Hispanic, and Asian) completed the protocols. All the participants were healthy and recreationally active, without any cardiovascular or metabolic diseases or physical disabilities. Participants were non-smokers and not taking any medications. Prior to the first visit, participants were screened for inclusion/exclusion criteria. Written informed consent form was obtained prior to participation. This study was approved by the Kean University Institutional Review Board for Human Subjects, IRB # FY2025-39.

### 2.2. Training Program

In this study, participants completed four visits to the Human Performance Laboratory. During the first visit, participants completed a written informed consent form, a health history questionnaire, a menstrual history questionnaire, a calcium intake questionnaire, and a bone-specific physical activity questionnaire (BPAQ). Participants’ anthropometric measurements were measured using a bioelectrical impedance analyzer (InBody770, Ltd., Seoul, Republic of Korea) and a wall-mounted stadiometer (Novel Products, Rockton, IL, USA).

### 2.3. Cardiorespiratory Fitness Assessment

Participants’ cardiorespiratory fitness was assessed using the Bruce protocol (Trackmaster, Full Vision, Inc., Newton, KS, USA). Participants performed the incremental test to volitional exhaustion, with treadmill speed and grade increased every three minutes according to the Bruce protocol. The test was terminated when the participant reached volitional fatigue and requested test termination. Maximal oxygen uptake (VO_2_max) was estimated from total exercise duration using previously validated equations [16].

### 2.4. Push-Up Test

Upper-body muscular endurance was assessed using the push-up test. Participants were instructed to remain in the standard down position with their hands pointing forward and directly under their shoulders, a straight back, and head up, using their toes as the pivotal point. Participants were then asked to lift their body by straightening their elbows, then return to the down position, stopping just before their chest touches the floor, and without their stomach touching the mat. The maximum number of push-ups the participant could perform in one minute without interruption, while maintaining proper form, was recorded. The test was terminated when the participant demonstrated visible straining or failed to maintain proper form for two consecutive repetitions [17].

### 2.5. Muscular Performance Assessment

The participants’ upper body strength was assessed by performing the handgrip strength test using a handgrip dynamometer (Takei Scientific Instruments, Yashiroda, Nigata, Japan). Each participant performed a handgrip strength assessment by flexing the elbow between 0–30° of flexion and 0–15° of ulnar deviation in both dominant and non-dominant hands. Participants were instructed to squeeze as hard as possible for 3–5 s, and the average of the three trials for each hand, separated by a 1 min rest, was used for data analysis. Participants’ lower-body muscular strength and power were assessed using the vertical jump test (Just Jump System, Probotics Inc., Huntsville, PA, USA) and the Tendo FitroDyne (Tendo Sports Machines, Trencin, Slovak Republic). Participants were instructed to step on the mat, stand with feet shoulder-width apart, and perform countermovement jumps. Participants completed three countermovement jumps, separated by a 1 min rest, and the data were averaged for analysis.

During the second visit, strength was evaluated using one-repetition maximum (1 RM) procedures for leg press, seated cable rows, barbell bench press, dumbbell deadlifts, and dumbbell seated shoulder press (Life Fitness and Hammer Strength LLC, Rosemont, IL, USA). Participants completed a 5 min low-intensity warm-up on a stationary bike, followed by 1–2 sets of light loads for each exercise. For each exercise, participants first performed 2 sets at 50% of their estimated 5–10 RM, with 1 min of rest between sets. Participants then performed 1 set at 75% of their estimated 5–10 RM with 2 min of rest between each set. The load was then gradually increased until participants reached their true 5–10 RM, performing a total of 3–5 maximal-effort sets [18].

### 2.6. Exercise Protocol

Participants completed the two exercise protocols (CT and TR) in random order, separated by a 2-week washout period. Both exercise protocols were performed early in the morning (07:00 h) after an overnight fast. Participants were instructed to maintain adequate hydration and consume a balanced meal no later than 20:00 h on the evening before the test session. Resting blood pressure was measured before both exercise protocols, and post-exercise blood pressure was measured after both protocols. Participants performed a five-minute warm-up on the aerobic bike, exceeding no more than 70 rpm. The CT protocol consisted of 3 rounds of 5 exercises: aerobic bike, push-ups, body-weight step-ups, Russian twists with a dumbbell, and front squats with a kettlebell. Each exercise was performed for one minute, with a 1 min rest between exercises and between rounds. RPE was taken after each round for the CT protocol only. The TR protocol consisted of 3 sets of 10 reps at 50–60% 1 RM, with one minute of rest between sets. The exercises included leg press, seated cable rows, barbell bench press, dumbbell deadlifts, and dumbbell seated shoulder press. The TR protocol was selected because it has been shown to elicit changes in sclerostin levels in young adults [19].

### 2.7. Blood Sampling and Biochemical Assays

**Figure 1:**
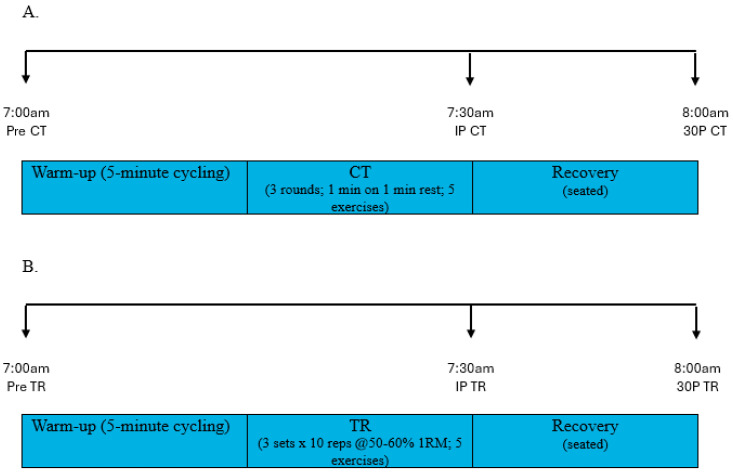
Timeline for blood draws for the CT (**A**) and TR (**B**) protocols.

Following an overnight fast for each protocol, acute exerkine markers were assessed at the same time of morning, starting at 7:00 a.m. For female participants, all blood samples were collected during the follicular phase of the menstrual cycle. Three venipuncture blood samples (7 mL) were taken by a nurse practitioner from an antecubital vein using a butterfly needle at rest before exercise (Pre), immediately post-exercise (IP), and 30 min post-exercise (30P) for both CT and TR protocols (Figure 1). After blood samples were collected, the blood was allowed to clot, then centrifuged (Eppendorf Centrifuge 5430, Hamburg, Germany), and the serum was transferred into microtubes and stored in a −80 °C freezer until the assays were performed. Samples were thawed only once, one hour prior to conducting the assays to avoid protein degradation. Commercial Enzyme-Linked Immunosorbent Assay Kits (ELISA) (Bio-Techne R&D Systems, Minneapolis, MN, USA) were used to measure serum levels of SCL, DKK-1, RANKL, OPN, BDNF, IL-6, and irisin (Elabscience Bionovation Inc., Houston, TX, USA). Step-by-step procedures were followed as per the assay kit instruction manuals and were performed in duplicates. In this study, the inter- and intra-assay CVs were less than 16%.

### 2.8. Statistical Analyses

Data were analyzed using IBM SPSS Statistics 31.0 (SPSS Inc., Chicago, IL, USA). All the descriptive data are reported as mean ± standard error. All the descriptive statistics were calculated for the dependent variables for each condition and time point. The Kolmogorov–Smirnov procedure was used to determine the normality of dependent variables. A two-way (protocol) × three (time point) repeated-measures ANOVA was used to assess the responses of SCL, RANKL, OPN, and irisin to CT and RT exercise protocols. When a significant protocol × time-point interaction occurred, the model was decomposed using a Bonferroni post hoc test for each condition. Independent variables were included in the stepwise regression models to determine whether any of them predict exerkines.

For the non-normally distributed data on BDNF, DKK-1, and IL-6, we evaluated the effects of sex, protocol, and time point using aligned rank transform (ART) mixed models in R (version 4.3.3; R Foundation for Statistical Computing, Vienna, Austria). ART mixed models were fitted for each marker to account for between-subject factors (Sex, Protocol) and a within-subject factor (Timepoint). For repeated measures, random intercepts for each participant, accounting for protocol and time point, were included.

For each marker, omnibus tests were computed for main and interaction effects. Type-III *χ*^2^ statistics were obtained from mixed-effects models. False discovery rate (FDR) correction using the Benjamini–Hochberg procedure was applied across markers for each effect to control for multiple comparisons. For effects that reached statistical significance in the omnibus tests, post hoc contrasts were performed with Bonferroni–Holm adjustment for multiple pairwise comparisons. Descriptive statistics were expressed as median (Q1, Q3) for each combination of Sex, Protocol, and Timepoint, due to the nonparametric nature of the ART procedure. For both normally and non-normally distributed data, the level of significance was set at *p* < 0.05.

## 3. Results

### 3.1. Participant Characteristics, Body Composition Variables, and Cardiorespiratory Fitness Assessment

We did not find any significant differences in any of the physical characteristic variables between male and female participants (Table 1). Female participants had higher post-exercise diastolic blood pressure than male participants (*p* = 0.017). Male participants had higher VO_2_max than female participants; however, no significant difference was observed (Table 2).

### 3.2. Lower and Upper Body Muscle Performance Assessment

We found a significant difference in right-hand grip strength between male and female participants (*p* = 0.004), but no differences in lower-body muscle performance variables. (Table 3). We also found a significant difference in 1 RM for seated cable rows, bench press, deadlifts, and seated shoulder press between male and female participants (*p* < 0.001) (Table 4).

### 3.3. Exerkines Responses Across Sex, Protocol, and Time Point

**Figure 2:**
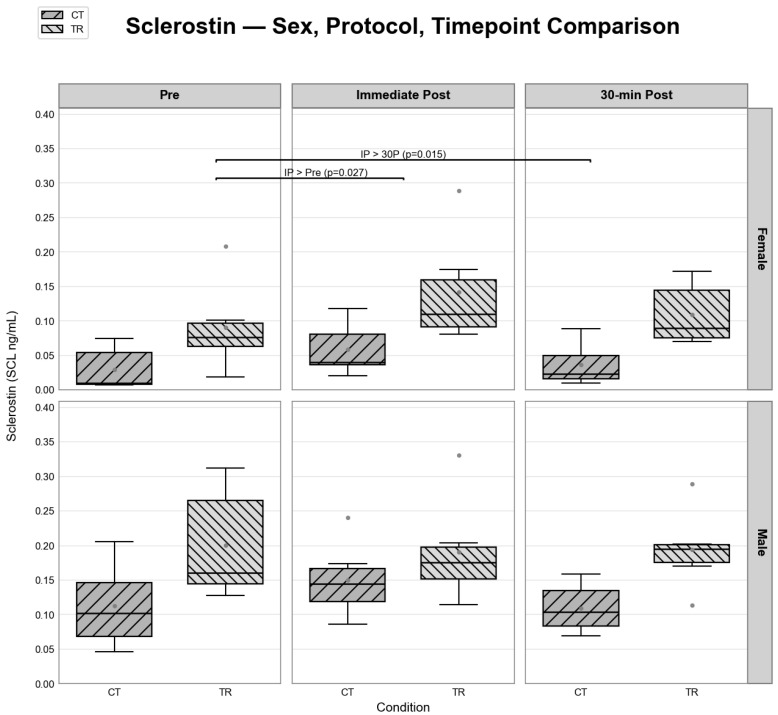
SCL concentrations for CT and TR protocol pre-exercise in male (n = 6) and female (n = 6) participants. (Mean ± 95% CI) significant *p* < 0.01 sex × protocol × time point effect.

**Figure 3:**
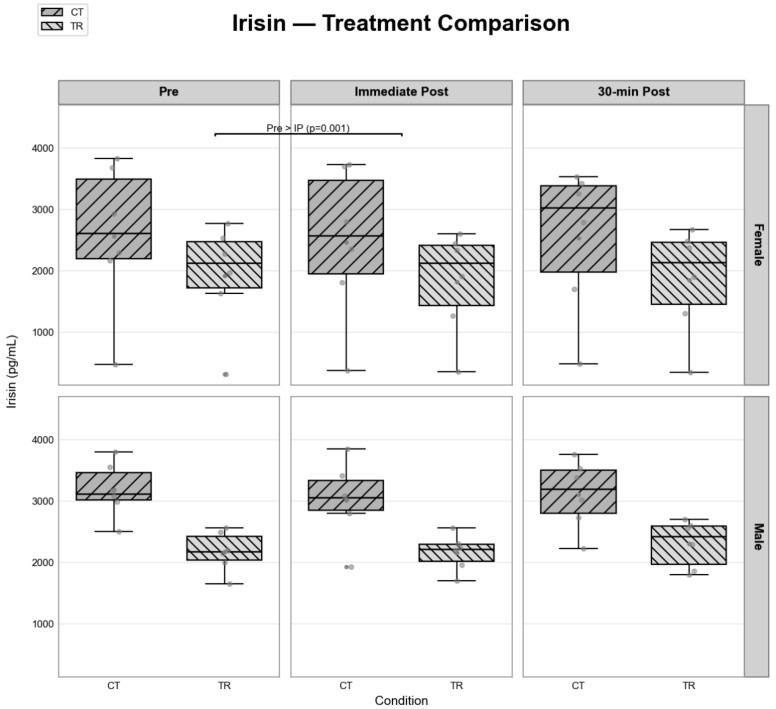
Irisin concentrations for CT and TR protocols in male (n = 6) and female (n = 6) participants. (Mean ± 95% CI) *p* < 0.01, a significant protocol × time point effect.

Figure 2 shows exerkine response across sex, protocol, and timepoint. There was a significant interaction between protocol, timepoint, and sex for SCL levels (*p* < 0.05). In males, SCL levels increased from Pre to IP under both training protocols (CT: 0.10 ± 0.02 ng/mL to 0.14 ± 0.02 ng/mL; TR: 0.20 ± 0.02 ng/mL to 0.21 ± 0.02 ng/mL). In both protocols, SCL levels decreased from IP to 30 P (CT: 0.14 ± 0.02 to 0.10 ± 0.01 ng/mL; TR: 0.22 ± 0.02 to 0.17 ± 0.02 ng/mL). In females, SCL levels increased from Pre to IP under both training protocols (CT: 0.03 ± 0.02 ng/mL to 0.06 ± 0.02 ng/mL; TR: 0.07 ± 0.02 ng/mL to 0.13 ± 0.02 ng/mL) (Table 5). There was a significant time effect for OPN (*p* = 0.01) and RANKL (*p* = 0.01) concentrations (Table 5). There were no significant differences between the sexes and protocols for OPN and RANKL. Marginal means for the time point showed that OPN was significantly higher at the Pre time point. Post hoc analyses showed that OPN levels decreased significantly from 30P to Pre (18.84 ± 0.92 to 15.69 ± 1.32 pg/mL) (*p* = 0.01). Similarly, RANKL showed a significant increase from Pre (0.38 ± 0.04 pg/mL) to 30P (0.57 ± 0.06 pg/mL) (*p* = 0.02); otherwise, there were no significant differences between protocols or sexes (Table 5). There was a significant main effect of protocol and time point on Irisin levels. Marginal means for the protocol and time point showed that Irisin levels were significantly higher in the CT protocol (2813.95 ± 288.63 pg/mL) compared to the TR protocol (2036.88 ± 198.67 pg/mL) (*p* = 0.001) (Table 5). Post hoc analyses showed that irisin levels decreased from Pre (28,761.73 ± 238.52 pg/mL) to IP (2364.85 ± 243.79 pg/mL) in both protocols (*p* = 0.01); however, we did not find significant differences between the two sexes (Figure 3).

Table 6 shows raw descriptive medians by marker, sex, protocol, and timepoint. BDNF levels were higher in the TR than in the CT protocol in both sexes. In males, rising from 0.479 pg/mL at Pre to 1.011 pg/mL at 30P, and in females, increasing from 0.464 to 0.960 pg/mL, compared to smaller increases during CT. DKK-1 was consistently higher during CT, with males showing stable values across time (1.276–1.294 pg/mL) and females reaching their highest medians at IP and 30P (1.723 and 1.581 pg/mL). IL-6 demonstrated the strongest protocol effect, with markedly higher concentrations during CT, males increased from 3.108 to 4.214 pg/mL and females from 4.103 to 4.396 pg/mL, while TR produced substantially lower values in both sexes. Overall, TR elicited greater BDNF responses, whereas CT produced higher DKK-1 and IL-6 values, with females generally showing higher concentrations across markers.

**Figure 4:**
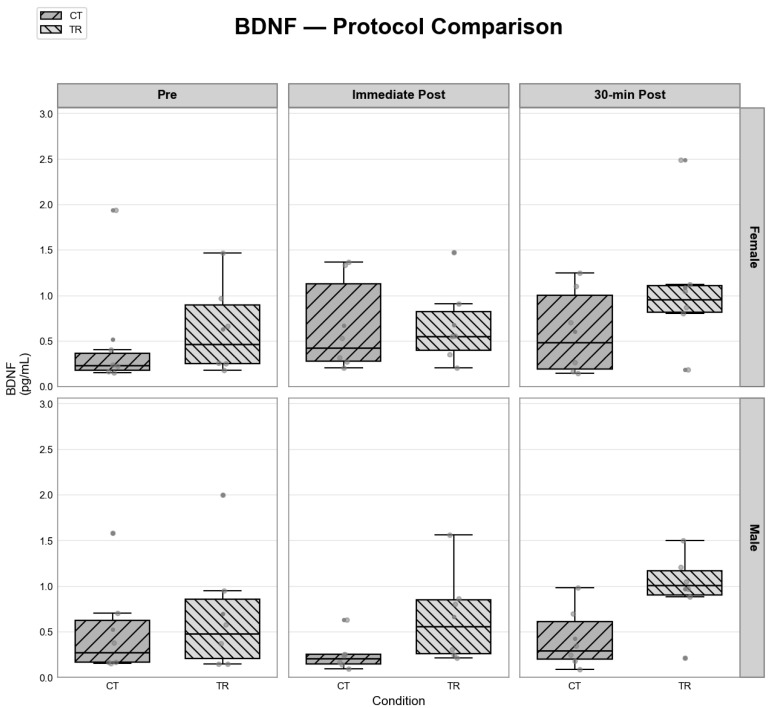
BDNF Post Hoc Comparison showing higher levels in TR protocol vs. CT. *p* < 0.01, a significant protocol main effect.

**Figure 5:**
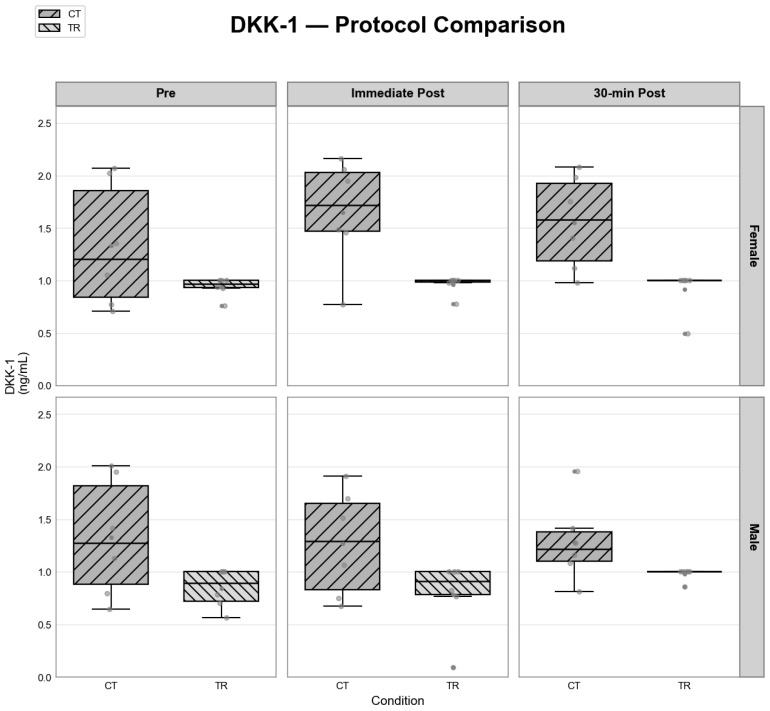
DKK-1 Post Hoc Comparison showing higher levels in CT protocol vs. TR. *p* < 0.01, a significant protocol main effect.

**Figure 6:**
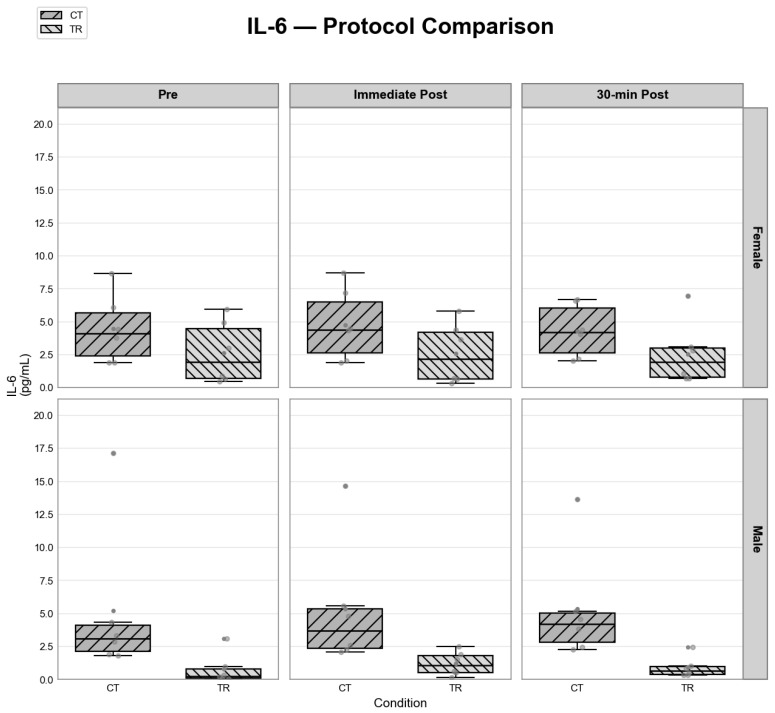
IL-6 Post Hoc Comparison showing higher levels in CT protocol vs. TR. *p* < 0.01, a significant protocol main effect.

For BDNF, Holm-adjusted post hoc comparison showed higher values in the TR condition than in the CT condition (*p* = 0.0101). For DKK-1 and IL-6, there were higher values in the CT condition DKK-1 (*p* < 0.001); IL-6: (*p* < 0.001) (Figure 4, Figure 5 and Figure 6).

Table 7 shows omnibus ART effects by marker. The ART mixed-model analyses revealed significant main effects of treatment for BDNF, DKK-1, and IL-6. Specifically, protocol exerted a strong effect on BDNF (χ^2^(1) = 7.15, *p* = 0.0075, FDR *p* = 0.010), DKK-1 (χ^2^(1) = 23.67, *p* < 0.001, FDR *p* < 0.001), and IL-6 (χ^2^(1) = 54.50, *p* < 0.001, FDR *p* < 0.001). No significant main effects of sex or timepoint were observed for BDNF, DKK-1, and IL-6 after FDR correction. Descriptive median (Q1, Q3) values by Sex, protocol, and time point for each dependent variable are provided in Table 6.

Table 8 shows regression analysis results for serum SCL, RANKL, OPN, BDNF, and IL-6. The regression analyses showed that several performance measures significantly predicted baseline exerkine levels across CT and TR conditions. For Pre CT SCL, push-ups (β = 0.006, *p* = 0.043), BDNF (β = 0.045, *p* = 0.002), and left handgrip strength (β = 0.002, *p* < 0.001) were significant predictors, explaining 94% of the variance (R^2^ = 0.94). For Pre TR SCL, JT TIA (β = 0.729, *p* = 0.019) and 1 RM bench press (β = 0.001, *p* = 0.006), with an R^2^ of 0.84, were significant predictors. Pre TR RANKL was predicted by TR protocol deadlifts (β = −0.009, *p* = 0.035), calcium intake (β = 0.000, *p* = 0.005), and JT TIA (β = −0.803, *p* < 0.001), accounting for 92% of the variance. Right-hand grip strength predicted pre-CT OPN (β = 0.215, *p* < 0.001; R^2^ = 0.85), while no significant predictors were found for pre-TR OPN or pre-CT BDNF. For Pre TR IL-6, calcium intake (β = 0.002, *p* = 0.01) and cBPAQ (β = 0.228, *p* = 0.001) significantly predicted pre-exercise levels during TR (R^2^ = 0.90). We did not find a significant predictor of performance measures for baseline irisin levels in either protocol.

## 4. Discussion

The results of this study revealed that acute bouts of CT and TR exercise programs elicited significant responses in exerkines. A strength of this study is the comparison of two ecologically valid and widely implemented training modalities, which enhances the real-world applicability and translational relevance of the findings. Furthermore, evaluating the broader range of exerkines provided deeper insight into bone–muscle crosstalk and the physiological mechanisms underlying exercise-induced adaptations.

The increased levels of SCL following an acute bout of exercise and the decrease at the 30 min post-exercise align with the established role of osteocyte-derived SCL in mechanotransduction mediated through the Wnt/β-catenin pathway [20], of which SCL and DKK-1 are the negative modulators of the Wnt/β-catenin pathway. Circulating SCL originates from osteocytes, and a previous study showed a positive correlation between SCL and bone marrow SCL expression, indicating that SCL reflects osteolytic production within bone tissue [21]. In existing literature, most studies have either examined physical activity levels in cross-sectional designs or controlled exercise interventions, and these studies have typically evaluated circulating SCL levels in only a single sex [22,23,24]. In this study, we found that SCL levels were elevated immediately post-exercise in both sexes but decreased thereafter. The possible mechanism with post-exercise increase could have contributed via plasma volume shifts, which we did not measure in this study. Further, reduced renal clearance could have transiently impaired SCL clearance from circulation, raising measured serum levels even without production. In animal models, mechanical loading downregulates SCL, and SCL is a key inhibitor of the Wnt/β-catenin pathway, which inhibits bone formation. Consequently, loading-induced SCL is the mechanism that stimulates osteogenesis [25]. However, in humans, SCL levels are elevated following aerobic exercise [19,26,27], which is consistent with our findings. In contrast, we also found a significant increase in TR exercise, unlike the previous findings [15,28]. These discrepancies across studies may reflect differences in loading magnitude, exercise training modalities, or mechanical strain characteristics. Notably, the significant decrease in SCL 30 min post-exercise in this study suggests a favorable acute adaptive response, similar to the study [19], which reported a significant decrease in SCL levels following 2 h post-exercise. Additionally, we also found higher SCL levels in males compared to females across protocols, which is also similar to the previous findings by [19], and potentially attributable to sex-related differences in bone mass and osteocyte activity; however, BMD was not measured in the present study, which limits our ability to compare across both sexes.

In the present study, circulating DKK-1 levels were significantly higher in the CT protocol compared to the TR protocol. Evidence suggests that exercise-induced changes in DKK-1 in the young healthy population remain limited. In a pediatric cohort, ref. [29] reported a significant decrease in DKK-1 levels 1 h after plyometric exercise. In contrast, ref. [30] found a significant increase in DKK-1 after 60 min post-exercise among military personnel. Similarly, ref. [31] reported a significant decrease in SCL and DKK-1 levels after 12 weeks of high-intensity interval training in patients with type II diabetes. In contrast, ref. [32], found no significant changes in DKK-1 levels among physically active women. An animal study reported that mechanical loading downregulates DKK-1 expression [33]. Compared with these studies, our study examined acute exercise responses in young healthy adults, highlighting that DKK-1 shows a protocol-dependent response even after a single bout of exercise. Moreover, participants’ characteristics such as training status and specific exercise modality might have contributed to these responses.

Findings on the effects of exercise on circulating OPN remain inconsistent across the literature. Some studies reported increased OPN levels after exercise [34,35]. We found OPN levels significantly decreased 30 min post-exercise, consistent with the findings by [36,37]. Notably, prior studies were conducted exclusively on obese participants. In contrast, our cohort consisted of non-obese individuals, which partly accounts for differences in OPN secretion and inflammatory and metabolic signaling pathways. OPN is a secreted matrix cell protein and is expressed in many cells, including bone and bone marrow mesenchymal stem cells, where it functions as an autocrine cytokine. Emerging evidence suggests OPN’s role in energy metabolism; however, the specific contribution of bone-derived OPN to metabolic regulation needs further investigation [38]. In animal models, OPN deficiency can delay muscle regeneration, suggesting that post-exercise OPN levels serve as a protective mechanism for muscle regeneration and warrant further investigation [39]. Several physiological factors may contribute to OPN’s responses in this study, including age, sex, training modality, and exercise-induced muscle hypoxia. Additionally, interactions with other immune mediators, such as interleukins, including IL-6, might have influenced OPN dynamics. Although we did not find a significant overall time effect in this study, the CT protocol elicited greater IL-6 responses compared to TR, which is inconsistent with the findings by [40]. In humans, studies have shown that IL-6 levels increase after acute exercise, possibly due to cellular stress that further activates MAPK and NFKB signaling in skeletal muscle. Regular exercise contributes to lowering the risk of metabolic disease, and the transient increase in circulating IL-6 might exert inhibitory actions on other pro-inflammatory cytokines, including TNF-α and IL-1β. However, this study did not analyze TNF-α. This IL-6-mediated anti-inflammatory cascade may prevent TNF-α-induced impairment of Glut-4 translocation and insulin transport in skeletal muscle, ultimately leading to metabolic dysfunction [41]. This suggests that the CT protocol could be valuable in sports science research to elicit both cardiovascular and muscular adaptations.

Interestingly, we also found that circulating RANKL levels increased 30 min post-exercise, which partially aligns with the previous findings by [42], who reported an acute increase in RANKL, 5 min following a single bout of high-intensity, low-impact exercise. Together, these findings support the idea that mechanical loading elicits a rapid osteoclast signaling response. In contrast, refs. [43,44] reported no change in RANKL levels following exercise. However, prior studies used chronic exercise protocol rather than acute exercise bouts, which may account for the variation in results. Evidence suggests that RANKL activity is tightly regulated by OPG, which inhibits pre-osteoclast differentiation by preventing RANK-RANKL binding, thus attenuating osteoclastic bone resorption. One plausible explanation for the post-exercise increase in RANKL observed in our study is that acute CT and TR exercise may transiently alter the OPG-RANKL signaling axis as part of the early phase of bone remodeling activation [45]. Further, high-intensity exercise is known to trigger a surge in pro-inflammatory cytokines and mobilize T and B cells that also secrete RANKL, potentially contributing to the elevated levels detected at 30 min post-exercise [46]. This mechanism further requires investigation to elucidate the acute osteo-immune response associated with different exercise modalities.

Our results showed that BDNF levels increased significantly in the TR protocol compared with the CT protocol, consistent with previous studies by [47,48,49]. The increase in BDNF levels after the TR protocol could partially explain the higher mechanical and metabolic demand associated with resistance loading. Mechanistically, resistance training is known to activate MAPK signaling, which might upregulate BDNF secretions [50,51]. Although BDNF is traditionally recognized for its role in neuroplasticity, emerging evidence indicates that its role extends beyond neural tissues. Notably, a recent in vitro study showed that BDNF-mediated activation of TrkB further stimulates PI3K/AKT signaling, promoting osteogenic differentiation and reducing cellular senescence in bone-derived mesenchymal stem cells [52]. Therefore, the increase in BDNF levels following the TR protocol in our study provides novel insight into its function as an exerkine with relevance not only to neural adaptation but also to bone remodeling. These findings support the growing concept of BNDF’s role in multiple signaling pathways and might represent a link between acute exercise loading and bone health. However, our results support the contention that exercise improves musculoskeletal health and may also promote improvements in brain health through BDNF-related mechanisms.

In the present study, circulating irisin levels were higher in the CT protocol than in the TR, and irisin levels decreased significantly immediately after exercise in both protocols, suggesting an acute exercise-induced modulation of circulating irisin. Although inconsistent with the previous findings [53,54], which showed a significant increase in irisin levels following a single bout of high-intensity training. Additionally, a meta-analysis by [55] reported an approximately 15% increase in irisin levels immediately after exercise, while also emphasizing that individual fitness status may exert a stronger influence on circulating irisin dynamics than the exercise modality per se. Furthermore, another study [56] reported a significant decrease in irisin levels after 16 weeks of training, suggesting that chronic exercise adaptations, rather than acute responses, may underlie changes in irisin dynamics. This study also highlights the association between body fat percentage and irisin levels, whereas no such association was observed in the present study. The discrepancy in findings across the studies may be attributed to exercise modality, duration, intensity, and participant characteristics, which warrants further investigation. One plausible explanation for the decrease in irisin levels immediately after exercise may be explained by the absence of acute changes in *FNDC5* mRNA expression, indicating that immediate irisin fluctuations are unlikely to reflect de novo synthesis [57]. Instead, circulating levels may be regulated predominantly by mechanisms of rapid release and clearance, and further research is needed to clarify these pathways.

This study has certain limitations that should be considered in future research. Despite the strengths of the crossover design, the small sample size (n = 12) and the use of a large effect size (Cohen’s d = 2.0) in the power calculation may overestimate the true magnitude of physiological responses among measured exerkines and may limit generalizability. Furthermore, the focus on acute exercise protocol impedes conclusions about chronic training adaptation. Future studies with larger cohorts are needed to validate these findings and refine effect size estimates across different exerkines. Plasma volume shifts were not measured, so some changes in exerkine levels may reflect exercise-induced fluid shifts rather than true secretion. This study included only a few exerkine panels, and future studies should include a broader array of bone turnover markers. Although exercise intensity was individualized based on 1 RM, differences in participants’ training backgrounds and strength could have influenced perceived effort. Finally, as all participants were healthy young adults meeting specific inclusion criteria, the findings may not generalize to older adults, clinical populations, or individuals with different fitness levels. Additionally, future studies should include bone mineral density assessment and dietary control to better explore the relationships between exerkines and bone health.

## 5. Conclusions

This pilot study showed time-dependent variations in exerkines following the acute bout of exercise. SCL levels increased immediately after exercise and subsequently decreased 30 min post-exercise in both exercise protocols, with notable sex differences observed. RANKL exhibited a delayed increase, with levels rising 30 min post-exercise in both protocols. Conversely, OPN levels decreased from pre-exercise to 30 min post-exercise. Irisin levels decreased only immediately after exercise in both protocols. BDNF, DKK-1, and IL-6 did not show significant changes across the three time points. SCL and BDNF levels were expressed higher in the TR protocol, whereas DKK-1, IL-6, and Irisin levels were expressed higher in the CT protocol. Overall, the findings suggest that SCL, RANKL, OPN, and irisin responded to the exercise bout, while the other exerkines did not show meaningful changes over time. In conclusion, both CT and TR training produced acute changes in circulating exerkines in young adults, with responses varying by exercise protocol. While these findings suggest a role for exercise modality in influencing bone–muscle crosstalk, they should be interpreted with caution given the study’s limitations. Further research in larger cohorts is required to confirm these observations.

## Figures and Tables

**Table 1:** Physical Characteristics and Body Composition Variables of Young Adults (Mean ± SE).

Variable	Male (n = 6)	Female (n = 6)
Age (yrs)	20.67 ± 1.02	20.17 ± 0.54
Height (cm)	169.76 ± 2.22	162.77 ± 3.32
Weight (kg)	83.58 ± 7.87	66.66 ± 7.37
Calcium Intake (mg/day)	527.67 ± 139.10	557.67 ± 135.75
pBPAQ	51.11 ± 9.80	29.99 ± 8.52
cBPAQ	5.89 ± 2.17	2.84 ± 1.08
tBPAQ	28.50 ± 4.92	16.42 ± 4.12
BMI	27.73 ± 3.20	24.68 ± 1.94
Body Fat %	20.98 ± 5.74	32.02 ± 4.05
Fat Mass (kg)	19.91 ± 6.87	22.61 ± 4.72
Fat Free Mass (kg)	38.01 ± 1.63	27.05 ± 2.53

pBPAQ = past Bone-Specific Physical Activity Questionnaire; cBPAQ = current Bone-Specific Physical Activity Questionnaire; tBPAQ = total Bone-Specific Physical Activity Questionnaire.

**Table 2:** Cardiorespiratory Fitness (Mean ± SE).

Variable	Male (n = 6)	Female (n = 6)
Push-up Score	30.33 ± 4.29	20.33 ± 1.52
RHR	72.00 ± 7.03	73.17 ± 3.25
RBP Systolic (mmHg)	119.17 ± 9.30	110.17 ± 3.12
RBP Diastolic (mmHg)	66.17 ± 3.24	66.00 ± 3.34
PEHR	129.00 ± 13.37	129.17 ± 5.40
PEBP Systolic (mmHg)	145.33 ± 13.30	150.33 ± 9.14
PEBP Diastolic (mmHg) *	60.17 ± 5.01	85.33 ± 7.26
VO_2max_ (mL/kg/min)	50.74 ± 3.47	43.41 ± 2.84
CTPEHR	115.66 ± 3.47	119.00 ± 14.26
TRPEHR	107.83 ± 19.48	109.16 ± 8.51

* *p* < 0.05 significant post-exercise diastolic BP; RHR = Resting heart rate; RBP = Resting blood pressure; PEHR = Post-exercise heart rate; PEBP = Post-exercise blood pressure; CTPEHR = Circuit training post exercise heart rate; TRPEHR = Traditional resistance post exercise heart rate.

**Table 3:** Upper and Lower Body Muscle Performance (Mean ± SE).

Variables	Male (n = 6)	Female (n = 6)
RHG **	39.89 ± 2.19	27.58 ± 2.57
LHG	37.55 ± 4.47	26.38 ± 2.63
TIA (s)	0.59 ± 0.04	0.50 ± 0.03
VJ Height (in)	17.85 ± 2.73	12.6 ± 1.59
Velocity	1.30 ± 0.09	1.18 ±0.05
Power (watts)	1023.94 ± 83.99	755.6 ± 86.68
Relative Power (kg/watts)	12.41 ± 0.84	11.37 ± 0.51

** p < 0.01 significant right-hand grip strength; RHG = Right-Hand Grip; LHG = Left-Hand Grip; TIA = Time in the air; VJ = Vertical Jump.

**Table 4:** One Repetition Maximum (1 RM) Testing (Mean ± SE).

Variable	Male (n = 6)	Female (n = 6)
Leg Press	514.00 ± 68.65	361.67 ± 66.60
Seated Cable Rows **	175.00 ± 9.22	98.33 ± 9.37
Bench Press **	180.00 ± 8.47	64.17 ± 6.38
Deadlifts **	98.33 ± 4.22	59.17 ± 5.39
Seated Shoulder Press **	56.67 ± 2.47	25.83 ± 3.00

** *p* < 0.01 significant weight for 1 RM for seated cable rows, bench press, deadlifts, and seated shoulder press.

**Table 5:** Exerkine Responses Across Sex, Protocol, and Timepoint (Mean ± SE).

Variables.	Male CT	Female CT	Male TR	Female TR	Significant Effects	*p*
SCL (ng/mL)						
Pre	0.11 ± 0.02	0.03 ± 0.02	0.21 ± 0.02	0.07 ± 0.02	Sex, Protocol, Time Point	0.008, 0.001, 0.04
IP	0.14 ± 0.02	0.06 ± 0.02	0.22 ± 0.02	0.13 ± 0.02	IP > Pre, 30P	0.027, 0.015
30P	0.10 ± 0.01	0.04 ± 0.02	0.17 ± 0.02	0.12 ± 0.02		
OPN (pg/mL)						
Pre	19.32 ± 1.8	19.39 ± 1.80	17.23 ± 1.43	19.40 ± 1.43	Time Point Pre > 30P	0.014 (0.04)
IP	14.98 ± 2.4	18.11 ± 2.42	15.62 ± 2.5	14.40 ± 2.56		
30P	16.31 ± 2.6	16.55 ± 2.60	13.05 ± 1.7	16.85 ± 1.70		
RANKL (pg/mL)						
Pre	0.35 ± 0.08	0.43 ± 0.08	0.31 ± 0.07	0.42 ± 0.07	Time Point	
IP	0.28 ± 0.07	0.35 ± 0.07	0.40 ± 0.08	0.29 ± 0.08	30P > Pre, IP	0.02, 0.001
30P	0.49 ± 0.08	0.51 ± 0.08	0.71 ± 0.12	0.59 ± 0.12		
Irisin (pg/mL)						
Pre	3018.65 ± 399.52	2734.61 ± 399.52	2091.57 ± 278.94	2002.11 ± 278.94	Protocol and time point	0.001
IP	2805.48 ± 430.28	2674.75 ± 430.28	2007.62 ± 275.32	1971.56 ± 275.32	Pre > IP	0.01
30P	2830.34 ± 408.03	2819.86 ± 408.03	2094.98 ± 297.85	2053.43 ± 297.85		

**Table 6:** Raw Descriptive Medians by Marker, Sex, Treatment, and Timepoint.

Marker	Sex	Treatment	Timepoint	N	Median (Q1, Q3)
BDNF (pg/mL)	Male	CT	IP	6	0.207 (0.141, 0.258)
	Male	CT	Post 30	6	0.297 (0.184, 0.702)
	Male	CT	Pre	6	0.276 (0.166, 0.707)
	Male	TR	IP	6	0.559 (0.245, 0.868)
	Male	TR	Post 30	6	1.011 (0.885, 1.211)
	Male	TR	Pre	6	0.479 (0.148, 0.955)
	Female	CT	IP	6	0.429 (0.271, 1.333)
	Female	CT	Post 30	6	0.486 (0.168, 1.101)
	Female	CT	Pre	6	0.233 (0.166, 0.406)
	Female	TR	IP	6	0.554 (0.354, 0.911)
	Female	TR	Post 30	6	0.960 (0.802, 1.127)
	Female	TR	Pre	6	0.464 (0.251, 0.972)
DKK-1 (ng/mL)	Male	CT	IP	6	1.294 (0.750, 1.697)
	Male	CT	Post 30	6	1.220 (1.090, 1.418)
	Male	CT	Pre	6	1.276 (0.799, 1.955)
	Male	TR	IP	6	0.917 (0.770, 1.006)
	Male	TR	Post 30	6	1.006 (1.006, 1.006)
	Male	TR	Pre	6	0.897 (0.705, 1.006)
	Female	CT	IP	6	1.723 (1.465, 2.063)
	Female	CT	Post 30	6	1.581 (1.120, 1.987)
	Female	CT	Pre	6	1.208 (0.774, 2.026)
	Female	TR	IP	6	1.006 (0.985, 1.006)
	Female	TR	Post 30	6	1.006 (1.006, 1.006)
	Female	TR	Pre	6	0.974 (0.933, 1.006)
IL-6 (pg/mL)	Male	CT	IP	6	3.689 (2.282, 5.576)
	Male	CT	Post 30	6	4.214 (2.476, 5.187)
	Male	CT	Pre	6	3.108 (1.905, 4.348)
	Male	TR	IP	6	1.073 (0.502, 1.894)
	Male	TR	Post 30	6	0.674 (0.352, 1.022)
	Male	TR	Pre	6	0.263 (0.083, 0.972)
	Female	CT	IP	6	4.396 (2.051, 7.211)
	Female	CT	Post 30	6	4.204 (2.178, 6.603)
	Female	CT	Pre	6	4.103 (1.923, 6.099)
	Female	TR	IP	6	2.159 (0.679, 4.385)
	Female	TR	Post 30	6	1.957 (0.712, 3.094)
	Female	TR	Pre	6	1.936 (0.637, 4.947)

**Table 7:** Omnibus ART Effects by Marker.

Variables	Effect	Test Value ^a^	Df	*p*-Value	Adj. *p*-Value (FDR ^b^)
BDNF (pg/mL)	Sex	0.3354737	1	0.562	0.594
	Protocol	7.1491876	1	0.0075 *	0.01 *
	Time point	4.1801622	2	0.124	0.247
	Sex: Protocol	0.1852342	1	0.667	0.667
	Sex: Timepoint	0.7422852	2	0.69	0.94
	Protocol: Timepoint	2.1499369	2	0.341	0.934
	Sex: Protocol: Timepoint	0.3303485	2	0.848	0.937
DKK-1 (ng/mL)	Sex	2.7546041	1	0.097	0.388
	Protocol	23.6687421	1	<0.001 **	<0.001 **
	Timepoint	1.5050720	2	0.471	0.617
	Sex: Protocol	1.7444637	1	0.187	0.667
	Sex: Timepoint	1.5087329	2	0.47	0.94
	Protocol: Timepoint	1.0482078	2	0.592	0.934
	Sex: Protocol: Timepoint	2.3590369	2	0.307	0.615
IL-6 (pg/mL)	Sex	0.5438761	1	0.461	0.594
	Protocol	54.5026553	1	<0.001 **	<0.001 **
	Timepoint	0.9664192	2	0.617	0.617
	Sex: Protocol	0.7270522	1	0.394	0.667
	Sex: Timepoint	0.5292637	2	0.767	0.94
	Protocol: Timepoint	0.1374544	2	0.934	0.934
	Sex: Protocol: Timepoint	0.1292577	2	0.937	0.937

^a^ Test = Chi-square; ^b^ FDR = Benjamini–Hochberg false discovery rate. * Significant at <0.01; ** Significant at <0.001.

**Table 8:** Regression analysis results for serum SCL, RANKL, OPN, BDNF, and IL-6.

Dependent Variable	Predictor Variables	β	SEE	R^2^	*p*
Pre CT SCL	Push-ups *	0.006	0.04	0.68	0.043
	BDNF C **	0.045	0.03	0.86	0.002
	Left HGS **	0.002	0.02	0.94	<0.001
Pre TR SCL	JT TIA *	0.729	0.05	0.67	0.019
	1 RM BB BP **	0.001	0.04	0.84	0.006
Pre TR RANKL	JT Velocity	0.603	0.15	0.38	0.245
	Trad DB DL *	−0.009	0.07	0.86	0.035
	CI **	0.000	0.06	0.92	0.005
	JT TIA **	−0.803	0.04	0.98	<0.001
Pre CT OPN	Right HGS **	0.215	1.92	0.85	<0.001
Pre TR OPN	tBPAQ	−0.104	1.63	0.85	0.363
Pre CT BDNF	CI	0.001	0.42	0.55	0.363
Pre TR IL-6	% BF	0.121	1.23	0.64	0.124
	CI *	0.002	0.99	0.79	0.01
	cBPAQ **	0.228	0.74	0.90	0.001

* p < 0.05; ** *p* < 0.01.

## Data Availability

We have included all the data from this study in tables and figures. Further inquiries can be directed to the corresponding author.
